# Effects of postmortem interval on myelin integrity in rhesus macaque brain tissue

**DOI:** 10.1111/bpa.70158

**Published:** 2026-10-16

**Authors:** Anna Novoseltseva, Rhiannon Robinson, Peter Niziolek, Allen Jang, Ting Xie, Ankita Tiwari, Ayman Abdelhakeem, Raymond Nicks, Yvonne Lucas, Qiong Yang, Farzad Mortazavi, David Boas, Hui Wang, Tara L. Moore, Douglas L. Rosene, Irving J. Bigio

**Affiliations:** ^1^ Department of Biomedical Engineering Boston University Boston Massachusetts USA; ^2^ Department of Anatomy and Neurobiology Boston University Boston Massachusetts USA; ^3^ Department of Psychological and Brain Sciences Boston University Boston Massachusetts USA; ^4^ Department of Electrical and Computer Engineering Boston University Boston Massachusetts USA; ^5^ Chobanian & Avedisian School of Medicine Boston University Boston Massachusetts USA; ^6^ School of Public Health Biostatistics Boston University Boston Massachusetts USA; ^7^ Department of Radiology, Athinoula A. Martinos Center for Biomedical Imaging, Department of Radiology Massachusetts General Hospital/Harvard Medical School Charlestown Massachusetts USA

**Keywords:** brain banking, polarization‐sensitive optical coherence tomography, postmortem interval, quantitative birefringence microscopy, rhesus macaque, scattering coefficient

## Abstract

When conducting microscopy of human brain sections, the postmortem interval (PMI) introduces myelin‐related artifacts that can confound neuropathological interpretation, yet the extent and detectability of these changes remain poorly characterized. This study examined the impact of controlled PMI on myelin integrity in rhesus macaque brain tissue using advanced optical imaging approaches. Brains from five aged rhesus macaques (20.5–28.5 years) were maintained at 4°C and analyzed at 0, 24, and 72 h postmortem. Myelin integrity in white and gray matter was assessed using quantitative birefringence microscopy to measure relative retardance and polarization‐sensitive optical coherence tomography to quantify scattering coefficients, retardance, and volumetric features. Increasing PMI was associated with progressive myelin degradation, reflected by significant reductions in relative optical retardance in white matter (*p* < 0.01) and gray matter (*p* < 0.001), alongside increased scattering coefficients in both regions (*p* < 0.001). Morphological analyses revealed a marked increase in myelin defects per mm^2^ with longer PMI (*p* < 0.001), characterized by swellings, vesicles, and structural discontinuities. These findings demonstrate that PMI causes significant detectable myelin changes, which can be quantified using optical imaging techniques, providing critical implications for interpreting postmortem neuropathological studies (especially with human brain tissues) and establishing a need for standardized PMI considerations in research protocols to ensure accurate pathological assessment in neurological disease investigations.

## Graphical abstract

In rhesus macaque brain, progressive myelin degradation over 72 h postmortem—with increasing numbers of myelin defects—altered myelin's optical properties, increasing scattering coefficient and decreasing relative retardance, as measured by quantitative birefringence microscopy and polarization‐sensitive optical coherence tomography.

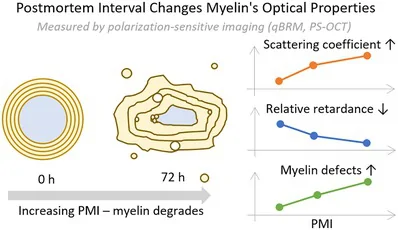

## 1 INTRODUCTION

Accurate neuropathological diagnosis relies on the ability to distinguish genuine disease‐related changes from postmortem artifacts. The postmortem interval (PMI)—the time between death and tissue fixation—varies widely in human studies and profoundly influences brain tissue integrity. The effects of PMI on brain tissue integrity are well‐established across multiple domains of neuroscience research. Comprehensive systematic reviews have documented that following death, brain cells rapidly undergo energy depletion and decomposition, with fluid shifts causing cell volume alterations and vacuolization as early events, while progressive loss of cell membrane structure occurs over periods ranging from several hours to several days [1]. These changes significantly impact the structural, molecular, and ultrastructural characteristics of brain tissue, creating substantial challenges for neuropathological interpretation [1, 2].

The assessment of myelin integrity is particularly critical for diagnosing demyelinating diseases such as multiple sclerosis [3] and age‐related neurodegeneration [4, 5]. However, PMI effects pose a fundamental challenge: distinguishing genuine pathological changes from postmortem artifacts. This challenge is particularly acute because human brain banking PMIs often substantially exceed those examined in controlled animal studies, frequently extending well beyond the 25‐h period typical of experimental investigations [6] and increasing the risk that PMI‐related artifacts may be misinterpreted as disease pathology. The problem is further compounded in aging populations, where PMI‐induced changes and age‐related myelin alterations may appear simultaneously [4, 5], making accurate pathological assessment exceptionally difficult.

At the cellular level, PMI effects begin within minutes after death, as cell membranes become non‐selectively permeable, permitting swelling and vacuolization [1]. Electron microscopy investigations have documented progressive myelin sheath degradation in nerve axons with increasing PMI, with more rapid deterioration at room temperature compared to 4°C storage [2, 7]. As decedents remain at room temperature for highly variable and inconsistently documented periods of time prior to refrigeration, this may be a significant variable to consider when assessing tissue integrity.

Molecular changes are equally significant, with proteins such as tubulin (critical for cytoskeletal structure) degrading significantly after 22 h without fixation, and ribonucleic acid (RNA) integrity decreasing with longer PMIs [6]. While the relationship between the cumulative effects of these cellular morphological changes and alterations in bulk tissue properties measurable by neuroimaging techniques remains to be established, an important step is to develop quantitative imaging modalities that are reliable and repeatable.

Magnetic resonance imaging (MRI) studies have demonstrated that PMI alters fundamental diffusion properties, with fractional anisotropy (FA) and diffusivity decreasing linearly in both white matter and gray matter, and artifacts appearing in T2‐weighted and diffusion tensor imaging after PMIs longer than 10 h [8, 9]. While MRI sensitively captures bulk tissue changes, it lacks the spatial resolution needed to assess myelin integrity at the single‐axon level, incentivizing the development of microscopic techniques.

Advanced polarization‐based label‐free optical imaging methods, including quantitative birefringence microscopy (qBRM) [10] and polarization‐sensitive optical coherence tomography (PS‐OCT) [11], have emerged as powerful tools for assessing myelin integrity by directly measuring the organizational structure of birefringent myelin sheaths [11, 12]. These techniques exploit myelin's inherent birefringence, arising from aligned macromolecular arrays of lipids and proteins, to provide quantitative metrics including relative retardance and scattering coefficients [13, 14]. Recent advances have demonstrated their capability for high‐resolution, label‐free imaging in human brain tissue, with PS‐OCT enabling volumetric mesoscale imaging of brain architecture and qBRM allowing single‐axon resolution assessment of myelin structural changes [15, 16].

Despite their promise, the effects of PMI on the optical properties measured by qBRM and PS‐OCT have not been systematically characterized. While PMI is known (from mostly qualitative studies) to degrade tissue quality for traditional histological, immunohistochemical, and neuroimaging assessments [6, 17], no studies have quantified how PMI alters relative retardance and scattering coefficients, the key parameters of these quantitative optical imaging techniques. This knowledge gap prevents researchers from distinguishing PMI‐induced artifacts from genuine pathological changes in postmortem myelin studies, limiting the interpretability of optical measurements in archived brain tissue.

Our study systematically characterizes how PMI affects the optical properties and morphological features measured by polarization‐based imaging techniques, using rhesus macaque brain tissue as a controlled model system, with properties similar to human tissues. By establishing quantitative baselines and quality‐control standards applicable across postmortem optical imaging studies, this work enables proper interpretation of myelin pathology in archived brain tissue and provides a framework for standardized PMI assessment in postmortem neuroscience research.

### 1.1 Hypothesis

In polarized‐light imaging, relative retardance reflects structural anisotropy in organized tissues, while scattering coefficients quantify light‐scattering probability [10], which increases with structural fragmentation and a higher density of optical interfaces.

We hypothesize that PMI‐induced myelin degradation will (1) reduce relative retardance by disrupting myelin's anisotropic organization and (2) increase scattering coefficients because of fragmentation and increased scatterer density (Figure 1). PS‐OCT and qBRM are optimal methods to test this hypothesis as they can quantify both parameters with high sensitivity.

**FIGURE 1:**
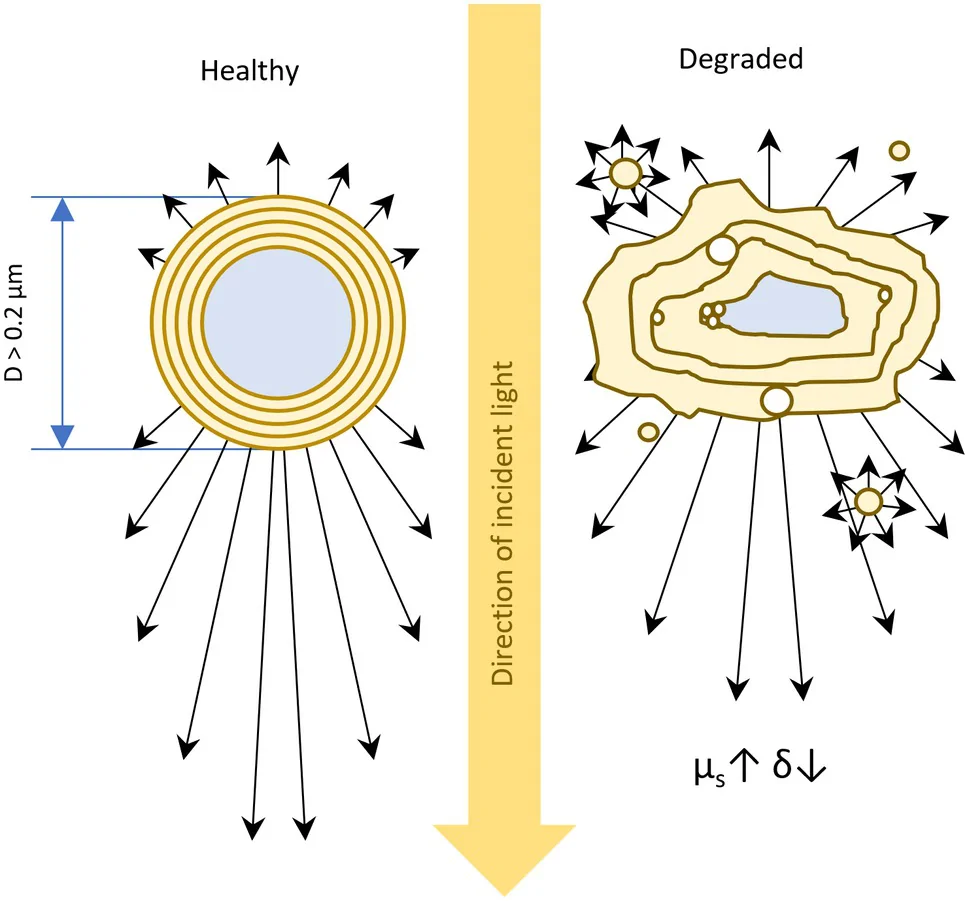
Schematic illustration of structural and optical changes in myelin during postmortem degradation. Left panel: Intact myelin sheath with organized lamellar structure exhibiting strong birefringence (high relative retardance and low scattering). Right panel: Degraded myelin showing structural disorganization, fragmentation, and increased number of scattering particles, resulting in decreased relative retardance and increased scattering coefficients. The degradation process creates smaller scattering particles while reducing structural anisotropy measurable by polarization‐sensitive imaging.

## 2 MATERIALS AND METHODS

This study employed a controlled experimental design to investigate PMI effects on myelin integrity using advanced optical imaging techniques. Detailed protocols for PS‐OCT and qBRM imaging systems have been previously published [16, 18]. Here, we describe the specific adaptations and protocols used for the PMI simulation study.

### 2.1 Experimental animals and study design

Brain tissue from five aged rhesus macaques (Macaca mulatta, 20.5–28.5 years) was utilized for this study (Table 1). All animals were subjects in our ongoing longitudinal aging studies (IACUC #00053). Tissue was maintained in Krebs buffer at 4°C and analyzed at three PMIs: 0, 24, and 72 h, simulating standard brain banking conditions, albeit with better temperature control.

**TABLE 1:** Sample demographics information.

Code	ID	PMI (h)	Sex	Age	Study
AM374	1	0, 24	M	28.5	Aging
AM395	2	0, 24, 72	F	23.5	Aging
AM406	3	24, 72	F	26.5	Aging
AM394	4	0, 24, 72	F	21.3	Neuroanatomy tracing
SM089	5	0, 24	F	20.5	Extracellular vesicles

Abbreviation: PMI, postmortem interval.

#### 2.1.1 Tissue harvesting and preparation

All animals were deeply anesthetized with intravenous propofol (2.5 mg/kg), followed by transcardial perfusion with ice‐cold Krebs buffer to clear the vasculature and halt cellular deterioration. The brain was immediately removed, blocked in situ, and 1.8‐cm coronal slabs were obtained from the occipital lobe for the current study.

Each slab was sectioned into six equal coronal sections: three designated for immersion fixation, cryoprotection, and subsequent cryostat sectioning for qBRM and immunohistochemistry, and three for immersion fixation and PS‐OCT imaging.

#### 2.1.2 Postmortem interval simulation

To isolate PMI‐related tissue degradation while maintaining experimental control, PMI was simulated ex vivo on unfixed tissue slabs maintained in Krebs buffer at 4°C for the designated PMI periods (0, 24, or 72 h). The 72‐h timepoint represents the upper bound of PMI commonly encountered in human brain banking. Following each PMI interval, tissue was then immersed in 4% paraformaldehyde at 4°C for 7 days of fixation. Post‐fixation, tissue designated for PS‐OCT imaging was transferred to fresh 4% paraformaldehyde to maintain optical clarity, while tissue designated for qBRM and immunohistochemistry was cryoprotected for frozen sectioning.

### 2.2 Multi‐modal processing pipeline

Post‐fixation tissue followed two parallel pathways (Figure 2). In the volumetric optical analysis pathway, tissue underwent phosphate‐buffered saline (PBS) washing, agarose embedding, serial‐sectioning, PS‐OCT imaging, followed by vibratome sectioning, cryosectioning, and qBRM imaging. In parallel, the high‐resolution validation pathway included cryoprotection, flash‐freezing, cryosectioning, immunohistochemistry, and confocal microscopy to provide molecular correlation with optical measurements.

**FIGURE 2:**
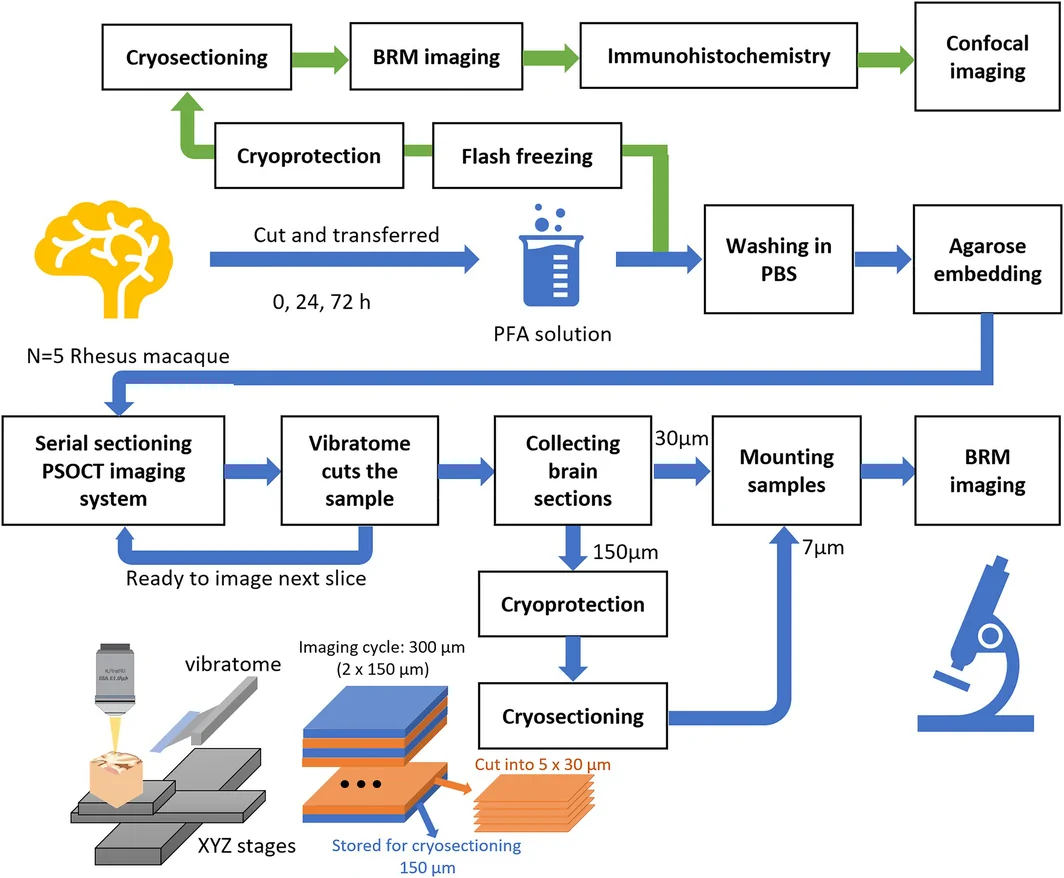
Multi‐modal experimental pipeline for quantifying postmortem interval (PMI) effects on myelin integrity. Brain tissue from five rhesus macaque underwent controlled PMI intervals (0, 24, and 72 h) at 4°C, followed by 4% paraformaldehyde fixation. The workflow splits into two pathways: (1) Immunohistochemical validation—cryoprotection, flash‐freezing, cryosectioning, quantitative birefringence microscopy (qBRM) imaging, immunohistochemistry, and confocal microscopy. (2) Volumetric optical analysis—phosphate‐buffered saline (PBS)‐washing, agarose embedding, serial‐sectioning polarization‐sensitive optical coherence tomography (PS‐OCT), followed by vibratome sectioning into either 150 μm sections (for white matter analysis—these sections undergo further cryoprotection and cryosectioning to 7 μm thickness for qBRM) or 30 μm sections (for direct gray matter qBRM imaging). This integrated approach enabled multi‐scale characterization from volumetric tissue properties to single‐axon resolution. BRM, birefringence microscopy.

Section thicknesses were optimized for each imaging modality: 150‐μm sections for PS‐OCT provide sufficient depth for volumetric analysis, while 7‐μm cryosections for qBRM minimize light‐scattering artifacts and enable high‐resolution myelin visualization for defect identification in high‐density white matter.

### 2.3 Serial‐sectioning polarization‐sensitive optical coherence tomography

#### 2.3.1 Sample preparation

Fixed tissue samples were washed in 0.01 M PBS containing 0.1% sodium azide for 7 days at 4°C to remove excess fixation agents. To minimize measurement variability, tissue samples from the same animal representing different PMI intervals (0, 24, and 72 h) were co‐embedded within a single block of 6‐mm^−1^ TiO_2_ agar (see Supporting Information S1: Additional File 1, Figure 2b). This co‐embedding strategy provides a critical methodological advantage: by maintaining all PMI timepoints from each animal within the same agar block with integrated calibration standards, inter‐block variability is eliminated, and measurement conditions are standardized, enabling direct quantitative comparison of PMI effects (Supporting Information S1: Additional File 1). Small regions of highly scattering agar (μ_s_ = 16 mm^−1^) were placed adjacent to tissue samples for surface detection and focus positioning.

#### 2.3.2 PS‐OCT imaging protocol

Imaging was performed using a serial‐sectioning PS‐OCT system as previously described [16], with 6‐μm axial and 5‐μm lateral resolution. Each scan captured volumetric information within the initial 150‐μm depth. Following complete surface scanning, samples were sectioned using a vibratome: 30‐μm sections for gray matter qBRM analysis, and 150‐μm sections for subsequent cryosectioning into 7‐μm slices for white matter qBRM analysis. Sectioned tissue was stored short‐term in PBS buffer solution containing 0.1% sodium azide at 4°C.

#### 2.3.3 Scattering coefficient analysis

Backscattering (μ_b_) and scattering coefficients (μ_s_) were extracted from reflectance signals using calibrated nonlinear fitting with a two‐step approach: (1) system parameter calibration using a TiO_2_ agar phantom with μ_s_ = 6 mm^−1^, and (2) tissue property extraction using calibrated parameters (detailed protocols in Supporting Information S1: Additional File 1).

#### 2.3.4 Retardance quantification

Relative retardance was extracted from PS‐OCT images and computed using the amplitudes of the two orthogonal polarization channels as previously described [16]. Individual imaging tiles were stitched using coordinates generated during volumetric reconstruction to create comprehensive relative retardance maps for region‐of‐interest analysis.

### 2.4 Quantitative birefringence microscopy

#### 2.4.1 Sample processing

For gray matter analysis, 30‐μm sections were mounted in 85% glycerol with 0.01% sodium azide. For white matter analysis, 150‐μm thick tissue samples were cryoprotected in 30% sucrose solution and sectioned at 7‐μm thickness using a cryostat at −20°C (see Supporting Information S2: Additional File 2).

#### 2.4.2 Circular‐polarization birefringence microscopy imaging

Qualitative birefringence imaging was performed using a modified slide scanner (VS‐120, Olympus) equipped with a narrowband filter (630 ± 10 nm) and circular polarizers as previously described [18]. Gray matter imaging utilized 20× magnification, while white matter analysis utilized 40× magnification to improve defect visualization in regions of high myelin density. Volumetric *z*‐stack images were acquired at 1‐μm intervals across full section thickness for three‐dimensional myelin architecture analysis.

#### 2.4.3 Quantitative birefringence microscopy

The qBRM system quantifies myelin structural organization by measuring transmitted light intensity as the incident polarization angle is rotated, followed by a circular‐polarization analyzing filter for detection (Figure 3A) [16]. Transmitted intensity varies sinusoidally with illumination polarizer angle, with amplitude changes reflecting myelin birefringence (Figure 3B).

**FIGURE 3:**
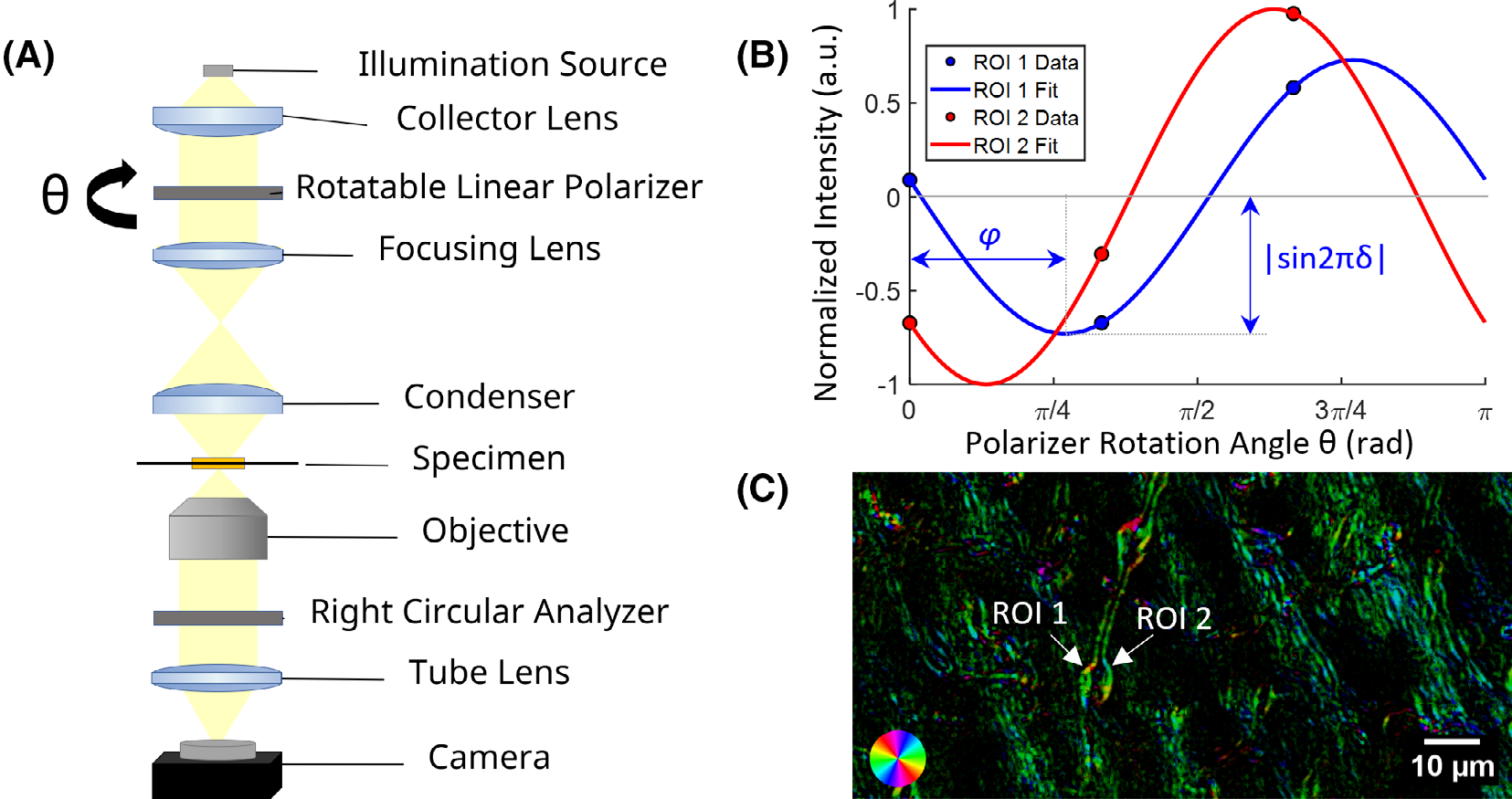
Quantitative birefringence microscopy system and measurement approach. (A) Optical configuration with rotatable linear polarizer allowing measurements at different polarization angles θ. System adapted from Blanke et al. [16]. (B) Measured light intensity from two tissue regions (region of interest [ROI] 1, ROI 2) varies sinusoidally with polarizer angle. Oscillation amplitude reflects relative retardance (|sin(2πδ)|); optic axis orientation indicated as φ. (C) Quantitative birefringence microscopy image showing myelin relative retardance combined with color‐coded fiber directions in rhesus macaque tissue. Colors indicate fiber orientation (color wheel). ROI 1 and ROI 2 mark measurement locations from panel (B). Scale bar: 10 μm.

Three parameters are extracted at each pixel using Equation (1):

(1) $$I\left(\theta \right)=\frac{I_{0}}{2}\left[1+\sin\left(2\left(\theta -\varphi \right)\right)\times \sin\left(2\pi \delta \right)\right].$$

#### 2.4.4 Relative retardance (|sin(2πδ)|)

Reflects the structural organization and density of myelin. This parameter is particularly sensitive to PMI‐induced degradation because myelin breakdown disrupts the parallel alignment of lipid bilayers, which generates the birefringence signal [12].

#### 2.4.5 Transmittance (*I*_0_)

Indicates overall light transmission through the tissue, reflecting tissue density and cellularity independent of myelin organization.

#### 2.4.6 Optic axis orientation (φ)

Maps the orientation of myelinated fibers (Figure 3C, color wheel).

All tissue sections were analyzed using the qBRM system at 4× magnification (Olympus MPlanFL NA = 0.13). Three slides per sample × PMI combination were measured to ensure comprehensive quantitative assessment across all experimental conditions, enabling morphological correlation with bulk tissue optical properties measured by PS‐OCT.

### 2.5 Morphological analysis and defect classification

#### 2.5.1 Region of interest selection

Gray matter and white matter regions were manually delineated based on PS‐OCT relative retardance maps [19], which clearly distinguish tissue boundaries because of differential myelin content. Mean relative retardance and scattering coefficient were calculated from 30‐μm (qBRM) or 150‐μm (PS‐OCT) sample volumes. Region of interest (ROI) locations were identified in qBRM images, where individual fiber orientation is directly visible, and then co‐registered to the corresponding PS‐OCT dataset to apply the same ROI for scattering coefficient and retardance measurements. White matter ROIs were selected as regions containing densely packed, parallel‐running myelinated fibers. Gray matter ROIs were selected within cortical sulci, where many myelinated fibers run in parallel, facilitating both quantitative optical measurement and visual identification of morphological defects.

#### 2.5.2 Morphological analysis workflow

Normal myelin in qBRM/birefringence microscopy (BRM) images appears as continuous, smoothly varying birefringent sheaths of consistent diameter running parallel to the section plane, interrupted only by nodes of Ranvier. Myelin defects were defined as any structural abnormality relative to this baseline pattern and classified into three subtypes: (1) vesicles—isolated, spherical birefringent shells visible in only one to two *z*‐stack planes, interpreted as detached myelin‐derived lipid debris; (2) swellings—an intact sheath that bulges outward, consistent with reported electron microscopy findings of granular, vacuole‐containing material accumulating beneath an otherwise continuous sheath [20]; and (3) delaminations/blebbings—defects in which the sheath itself splits or fragments. Fiber orientation, visualized as a color‐coded map (plotting φ), was used only to help locate candidate regions for closer inspection and was not itself a classification criterion; an orientation shift alone, without an accompanying morphological change meeting one of the criteria above, was not classified as a defect. Because annotation was performed on full three‐dimensional *z*‐stacks (1‐μm step size) rather than single two‐dimensional images, annotators tracked each candidate structure across neighboring *z*‐planes to distinguish a true isolated vesicle (visible in only one to two planes) from two adjacent, obliquely‐sectioned axons, which instead persist as separate elongated profiles across several planes.

Each annotator scored the complete dataset independently, without consulting the others, to minimize inter‐rater bias; structures were classified as “mixed” when they displayed overlapping features of more than one subtype (e.g., a swelling with partial delamination) or when annotators could not otherwise reach consensus. These classification criteria were developed and validated in collaboration with neuropathologists and are described in further detail in our related work [18]. For summary reporting, “total defects” refers to the sum of vesicles, swellings, delaminations/blebbings, and mixed‐type defects within a given region of interest.

#### 2.5.3 Consensus annotation approach

Three independent annotators, blinded to PMI condition, examined the entire dataset to ensure robust defect identification. A consensus approach [17] was implemented where only defects identified by at least two annotators were considered true positives, employing an algorithm requiring a minimum of 20% intersection over union (IoU) with ±1 *z*‐plane tolerance [18].

### 2.6 High‐resolution validation

#### 2.6.1 Cryoprotection

To prevent unnecessary tissue damage during freezing and thawing of tissue, all tissue is cryoprotected prior to flash‐freezing. Once fixation is complete, tissue is immersed in 10% glycerol in 2% dimethyl sulfoxide (DMSO) for 24 h, or until sinking (whichever is longer). The tissue is transferred into a vial with 20% glycerol in 2% DMSO, and immersed for another 24 h, or until sunk. Finally, tissue is immersed in 30% glycerol in 2% DMSO and immersed for 7 days. All cryoprotection occurs at 4°C.

#### 2.6.2 Flash‐freezing

Once cryoprotected, tissue is flash‐frozen in a double‐boiler setup of 2‐methylbutane surrounded by dry ice in 100% isopropyl alcohol. When the 2‐methylbutane solution reaches −75°C, tissue is rapidly lowered into the solution and held immersed for 30 min, while monitoring to ensure the temperature does not exceed −70°C. When frozen, tissue is immediately wrapped in aluminum foil and put into a −80°C freezer for storage.

#### 2.6.3 BRM imaging

Imaging was performed using the custom system described by Blanke et al. [21] at 40× magnification. Volumetric *z*‐stack images were acquired at 1‐μm intervals across the full section thickness. Slide coordinates were recorded to enable co‐registration with subsequent immunohistochemical staining on identical tissue regions.

#### 2.6.4 Immunohistochemistry protocol

Immunohistochemical processing was performed on slides previously imaged with qBRM. Coverslips were carefully removed while immersed in 0.05 M Tris‐buffered saline (TBS). Tissue sections underwent the following treatment sequence: washing in 0.05 M TBS, washing with 0.5% Triton X‐100 in Superblock®, and incubation with primary antibodies in 0.05 M TBS.

#### 2.6.5 Primary and secondary antibodies

Primary antibodies included degraded myelin basic protein (dMBP) and axonal neurofilaments (SMI312), each applied at 1:500 dilution. Tissue was incubated at room temperature for 3 h, then at 4°C for 48 h. Following TBS washing (3 h), secondary antibodies (Alexa Fluor Plus 555 and 488) were applied with incubation at room temperature for 8 h, then overnight at 4°C. Final washing (6 h) was followed by rapid drying, followed by application of mounting media (85% glycerol) and cover‐slipping for confocal microscopy at 10× (whole hemisphere) and 40× (regions of interest) magnifications.

### 2.7 Statistical analysis

Statistical analyses were performed using IBM SPSS Statistics version 27. Data are presented as mean ± standard deviation unless otherwise specified.

#### 2.7.1 Mixed‐effects modeling

Linear mixed‐effects models were fitted with PMI (0, 24, and 72 h) as a fixed categorical factor and animal ID as a random intercept to account for repeated measures. Separate models were constructed for scattering coefficients, relative retardance values, and defect counts across gray matter and white matter regions. Model assumptions were evaluated using residual diagnostics.

#### 2.7.2 Inter‐annotator agreement assessment

Inter‐annotator reliability for myelin defect identification was assessed using intraclass correlation coefficients (ICC) with a two‐way mixed‐effects model with consistency definition; appropriate for fixed raters and relative consistency assessment. Both single‐rater and average‐rater reliability were calculated with 95% confidence interval (CI) for total defect counts and individual defect subtypes, computed separately for gray matter (*n* = 100 images) and white matter (*n* = 100 images).

#### 2.7.3 Correlation analysis

To examine associations between optical properties and structural changes independent of PMI effects, Pearson correlations were computed between residuals from linear mixed‐effects models fitted for each optical property (scattering coefficient, relative retardance, and defect count). This residualization approach controls for systematic PMI effects while preserving within‐subject covariation patterns. Correlations were computed separately for white matter and gray matter because of their distinct optical properties.

#### 2.7.4 Multiple comparisons

Pairwise comparisons between PMI timepoints used estimated marginal means with least‐significant‐difference adjustment. Models employed maximum likelihood estimation with 95% CIs, 100 maximum iterations, and absolute convergence criteria of 0.000001. Model fit was evaluated using Akaike information criterion (AIC), Bayesian information criterion (BIC), and residual diagnostics. Statistical significance was set at *p* < 0.05. As a sensitivity analysis, defect counts were additionally analyzed using two‐way analysis of variance (ANOVA) (animal and PMI as factors) with Tukey's Honesty Significant Difference (HSD) and Bonferroni‐corrected post hoc comparisons.

#### 2.7.5 Effect sizes and statistical power

Cohen's *d* effect sizes were calculated to quantify the magnitude of PMI effects relative to measurement precision. Statistical power was computed using pooled standard deviations across PMI intervals and within‐subject variability, demonstrating adequate sensitivity for detecting biologically meaningful changes.

## 3 RESULTS

### 3.1 PMI‐induced changes in optical scattering properties

PS‐OCT analysis revealed progressive, statistically significant increases in scattering coefficients (μ_s_) with increasing PMI in both gray and white matter regions across all five rhesus macaque samples (Figure 4). In white matter, mean scattering coefficients increased from 10.4 ± 1.5 mm^−1^ at 0 h PMI to 15.4 ± 1.2 mm^−1^ at 72 h (*p* < 0.001, *n* = 5 animals). Gray matter regions demonstrated an even more pronounced increase, with μ_s_ values rising from 5.88 ± 0.76 mm^−1^ at 0 h to 9.93 ± 0.81 mm^−1^ at 72 h PMI (*p* < 0.001, *n* = 5 animals).

**FIGURE 4:**
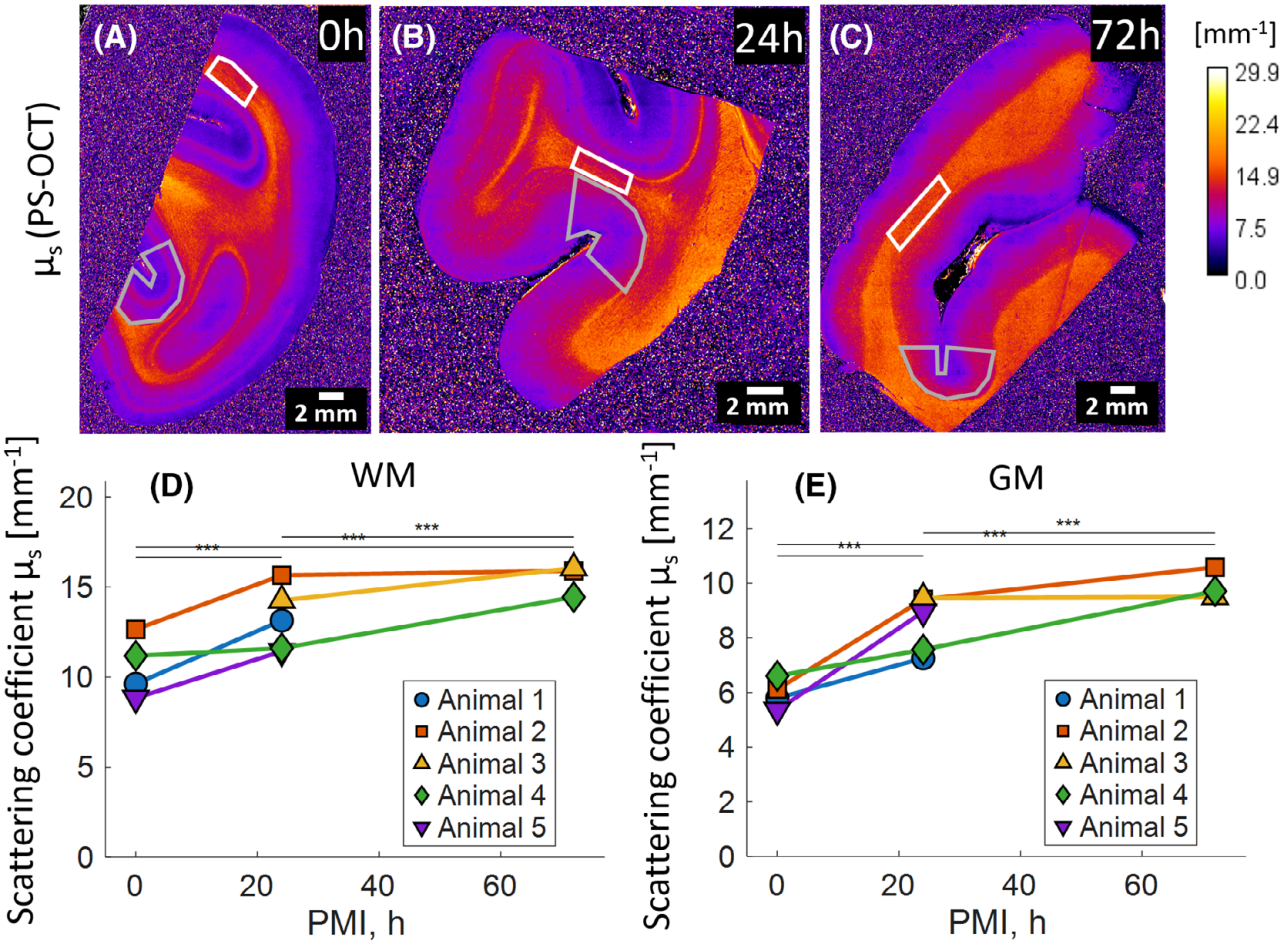
Scattering coefficient measurements across postmortem interval (PMI) intervals. (A–C) Representative scattering coefficient (μ_s_, mm^−1^) maps at 0, 24, and 72 h PMI. Gray outlines indicate gray matter regions of interest (ROIs) and white outlines indicate white matter ROIs. (D) White matter (WM) scattering coefficients increased progressively with PMI across all five animals. (E) Gray matter (GM) scattering coefficients showed similar PMI‐dependent increase. Lines connect individual animal values across PMI intervals. Statistical comparisons were performed using linear mixed‐effects models with PMI as a fixed factor and animal ID as a random intercept; pairwise comparisons between PMI timepoints used estimated marginal means with least‐significant‐difference adjustment. Statistical significance: ***p < 0.001. Scale bar: 2 mm. PS‐OCT, polarization‐sensitive optical coherence tomography.

Effect size analysis quantified the magnitude of PMI‐induced changes in scattering coefficients. Cohen's *d* values ranged from 1.63 to 4.39 across tissue types and PMI intervals, indicating very large standardized effects. Statistical power exceeded 99% with *n* = 5 animals. Within‐subject measurement precision was 1.12 mm^−1^, substantially smaller than the observed PMI‐induced changes.

### 3.2 Progressive loss of relative Retardance with PMI

Relative retardance measurements demonstrated significant PMI‐dependent changes in both tissue types (Figure 5). PS‐OCT measurements in white matter showed a progressive decline from (0.87 ± 0.12)·10^−3^ at 0 h PMI to (0.55 ± 0.04)·10^−3^ at 72 h PMI (*p* < 0.001, *n* = 5 animals). qBRM measurements in gray matter revealed a significant early decline followed by stabilization, with relative retardance decreasing from 0.056 ± 0.016 at 0 h to 0.041 ± 0.008 at 24 h (*p* = 0.001), then plateauing at 0.035 ± 0.014 by 72 h PMI (24 h vs. 72 h: *p* = 0.191; overall PMI effect: *p* < 0.001, *n* = 5 animals).

**FIGURE 5:**
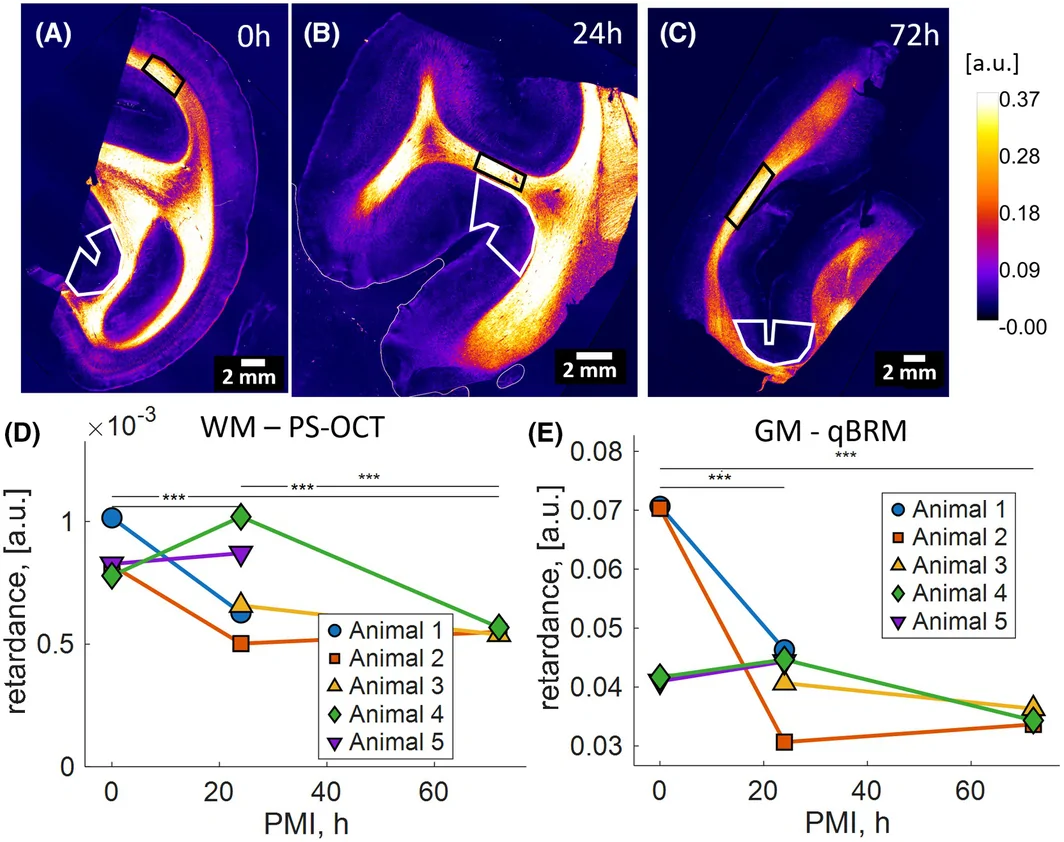
Relative retardance measurements across postmortem interval (PMI) intervals. (A–C) Representative quantitative birefringence microscopy (qBRM) relative retardance maps for 0, 24, and 72 h PMI showing progressive signal decrease (darker regions indicate lower relative retardance). Black outlines indicate white matter regions of interest (ROIs), white outlines indicate gray matter ROIs. (D) Polarization‐sensitive optical coherence tomography (PS‐OCT) measurements in white matter (WM) show significant decrease in relative retardance with PMI. (E) qBRM measurements in gray matter (GM) show decrease in relative retardance with PMI. Lines connect individual animal values across PMI intervals. Statistical comparisons were performed using linear mixed‐effects models with PMI as a fixed factor and animal ID as a random intercept; pairwise comparisons between PMI timepoints used estimated marginal means with least‐significant‐difference adjustment. Statistical significance: ***p < 0.001. Scale bar: 2 mm.

PS‐OCT measurements in gray matter showed increased relative retardance at longer PMI intervals, contrasting with the decreased retardance observed in white matter and in gray matter qBRM measurements.

### 3.3 Morphological analysis of PMI‐induced myelin defects

High‐resolution BRM imaging enabled morphological characterization of PMI‐induced myelin structural changes at the single‐axon resolution (Figures 6 and 7). Manual annotation revealed a progressive increase in defect density with increasing PMI.

**FIGURE 6:**
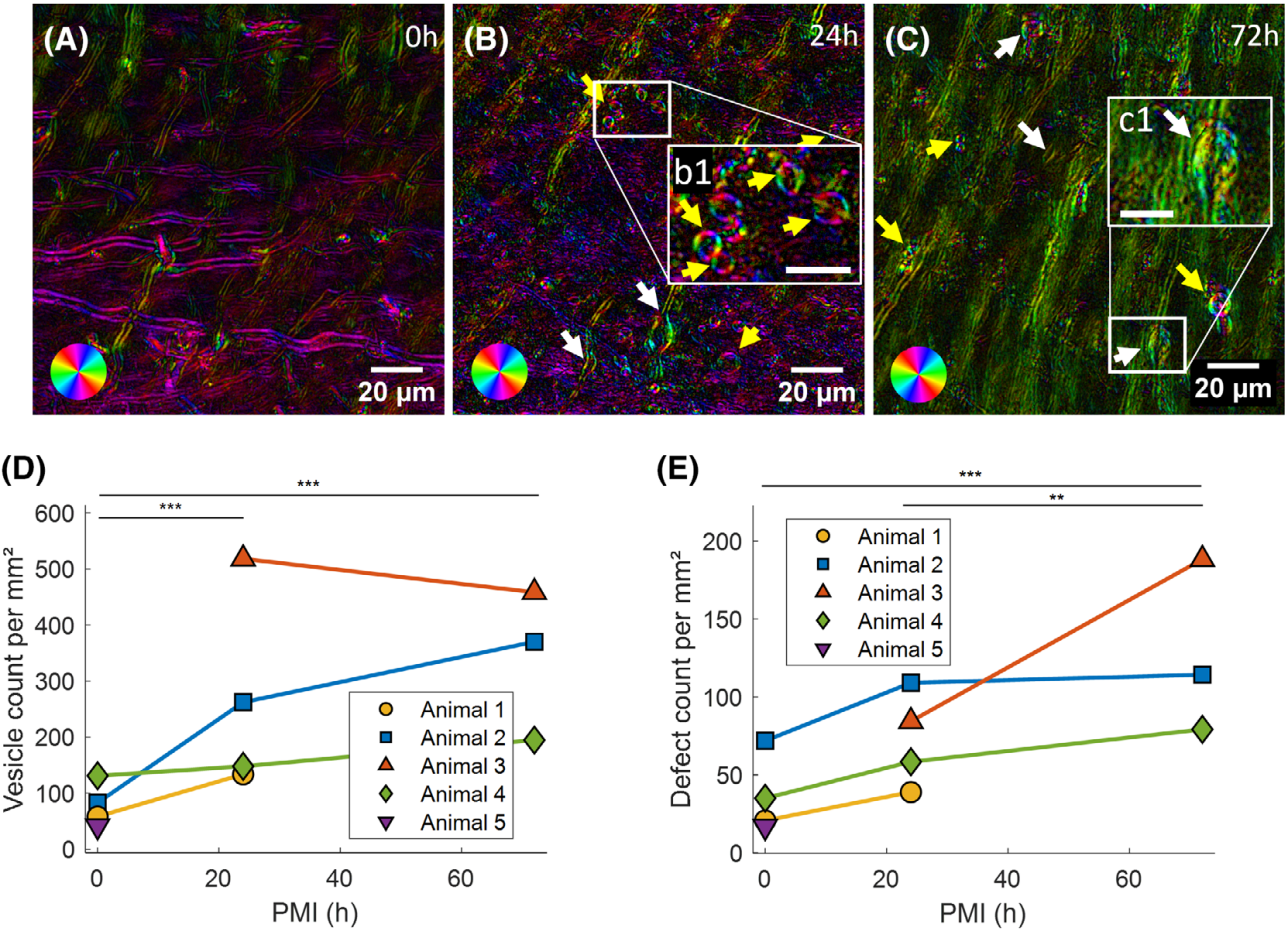
Representative quantitative birefringence microscopy images showing myelin structural changes in gray matter. (A–C) Gray matter at 0, 24, and 72 h postmortem interval (PMI) with optic axis orientation colormap. Defects highlighted were randomly selected from the annotated dataset; arrow color indicates defect subtype (yellow = vesicle, white = swelling). (B1, C1) Magnified views of boxed regions showing defect detail. (D) Vesicle density showing progressive increase with PMI. (E) Combined swelling and delamination/blebbing defect density showing PMI‐dependent increase. Lines connect individual animal values across PMI intervals. Statistical comparisons were performed using linear mixed‐effects models with PMI as a fixed factor and animal ID as a random intercept; pairwise comparisons between PMI timepoints used estimated marginal means with least‐significant‐difference adjustment. Statistical significance: ***p < 0.001, **p < 0.01. Scale bars: 20 μm (A–C), 10 μm (B1, C1).

**FIGURE 7:**
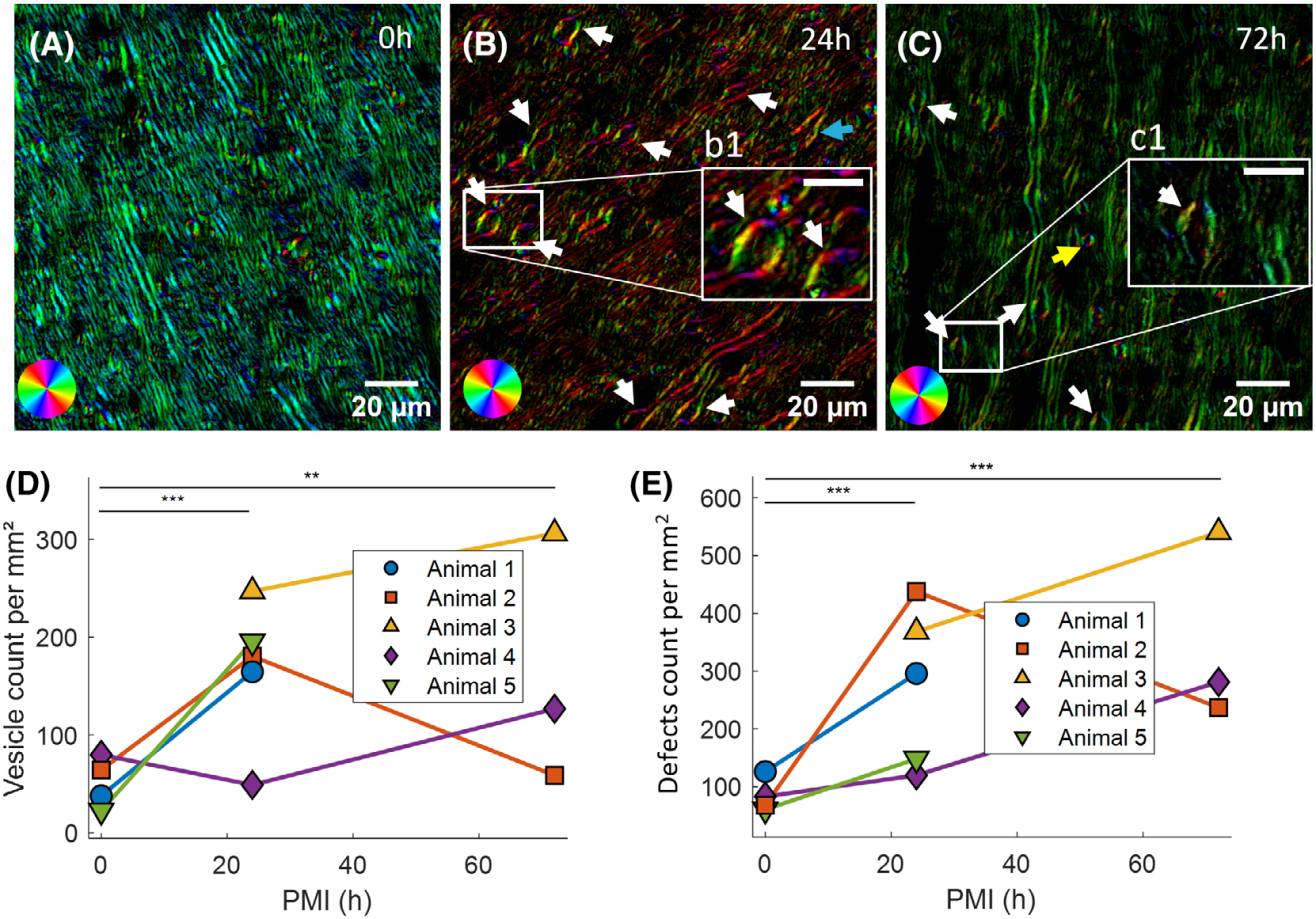
Quantitative birefringence microscopy analysis of white matter regions showing postmortem interval (PMI)‐dependent myelin degradation. (A–C) Representative white matter images at 0, 24, and 72 h PMI using optic axis orientation colormap. Defects highlighted were randomly selected from the annotated dataset; arrow color indicates defect subtype (yellow = vesicle, white = swelling, blue = delamination/blebbing). Color‐wheel shading reflects fiber orientation only, including at paranodes and normal fiber curvature, and does not itself denote a defect; classification criteria are described in Section 2. (B1, C1) Magnified views of boxed regions showing defect detail. (D) Vesicle density analysis showing increase with PMI in white matter regions. (E) Combined swelling and delamination/blebbing defect density analysis showing PMI‐dependent degradation increase. Lines connect individual animal values across PMI intervals. Statistical comparisons were performed using linear mixed‐effects models with PMI as a fixed factor and animal ID as a random intercept; pairwise comparisons between PMI timepoints used estimated marginal means with least‐significant‐difference adjustment. Statistical significance: ***p < 0.001, **p < 0.01. Scale bars: 20 μm (A–C), 10 μm (B1, C1).

In gray matter (Figure 6), total myelin defect counts increased significantly from 150 ± 86 defects/mm^2^ at 0 h PMI to 484 ± 372 at 24 h, and 629 ± 293 at 72 h PMI (*p* < 0.001, *n* = 5 animals). Individual defect subtypes showed similar temporal patterns, with vesicle counts rising from 77 ± 57 at 0 h to 341 ± 160 at 72 h PMI (*p* < 0.001). Mixed‐type defects peaked at 24 h (145 ± 159) before stabilizing at 72 h (160 ± 105, *p* = 0.010).

White matter regions demonstrated greater defect accumulation (Figure 7), with total defect counts rising from 136 ± 38 defects/mm^2^ at 0 h to 442 ± 191 at 24 h, and 517 ± 291 at 72 h PMI (*p* < 0.001, *n* = 5 animals). White matter showed distinct temporal patterns for individual defect types: vesicle counts increased from 51 ± 26 to 164 ± 128 (*p* < 0.001), while combination of swelling and delamination/blebbing defects showed progressive increases from 85 ± 29 to 353 ± 164 over the same PMI interval (*p* < 0.001). Immunohistochemical labeling confirmed progressive structural disruption across PMI timepoints (see Supporting Information S3: Additional File 3). An additional sensitivity analysis (using two‐way ANOVA with Tukey's HSD and Bonferroni correction) showed that the overall PMI effect remained significant for all four defect measures (gray matter vesicles: *F*(2,93) = 9.12, *p* < 0.001; gray matter swelling/delamination defects: *F*(2,93) = 5.78, *p* = 0.004; white matter vesicles: *F*(2,93) = 10.04, *p* < 0.001; white matter swelling/delamination defects: *F*(2,93) = 18.73, *p* < 0.001), and all pairwise comparisons between 0 h and later PMI timepoints remained significant under both corrections (*p* ≤ 0.047), consistent with our primary linear mixed‐effects model (Supporting Information S4: Additional File 4).

### 3.4 Classification and distribution of myelin defect types

The distribution of defect types differed markedly between tissue regions. In gray matter, vesicles comprised 54.2% of total defects, followed by mixed‐type defects (25.4%) and combination of swelling and delamination/blebbing defects (20.4%). White matter showed a more balanced distribution: combination of swelling and delamination/blebbing comprised 36.7%, vesicles 35.1%, and mixed‐type defects (28.2%).

Mixed defects, where annotators could not reach unanimous classification, comprised 25.4% of gray matter and 28.2% of white matter defects.

### 3.5 Inter‐annotator agreement

Inter‐rater consistency was tissue‐specific. Gray matter images demonstrated good agreement (ICC = 0.756, 95% CI: 0.660–0.829), while white matter annotation showed poor‐to‐fair agreement (ICC = 0.443, 95% CI: 0.223–0.608). To address lower reliability, consensus annotation was employed requiring agreement between at least two annotators.

### 3.6 Correlation between optical properties and morphological changes

To examine relationships between optical measurements and structural changes, Pearson correlations were computed using residuals from mixed‐effects models.

Scattering coefficient showed positive correlation with total defect density in gray matter (*r* = 0.676, *p* = 0.016) and white matter (*r* = 0.741, *p* < 0.006) (Figure 8A,B). qBRM relative retardance showed negative correlations with defect density in gray matter (*r* = −0.643, *p* = 0.024) and white matter (*r* = −0.388, *p* = 0.212) (Figure 8C,D). In white matter, PS‐OCT scattering coefficient showed a strong negative correlation with relative retardance (*r* = −0.558, *p* < 0.001) (Figure 8E).

**FIGURE 8:**
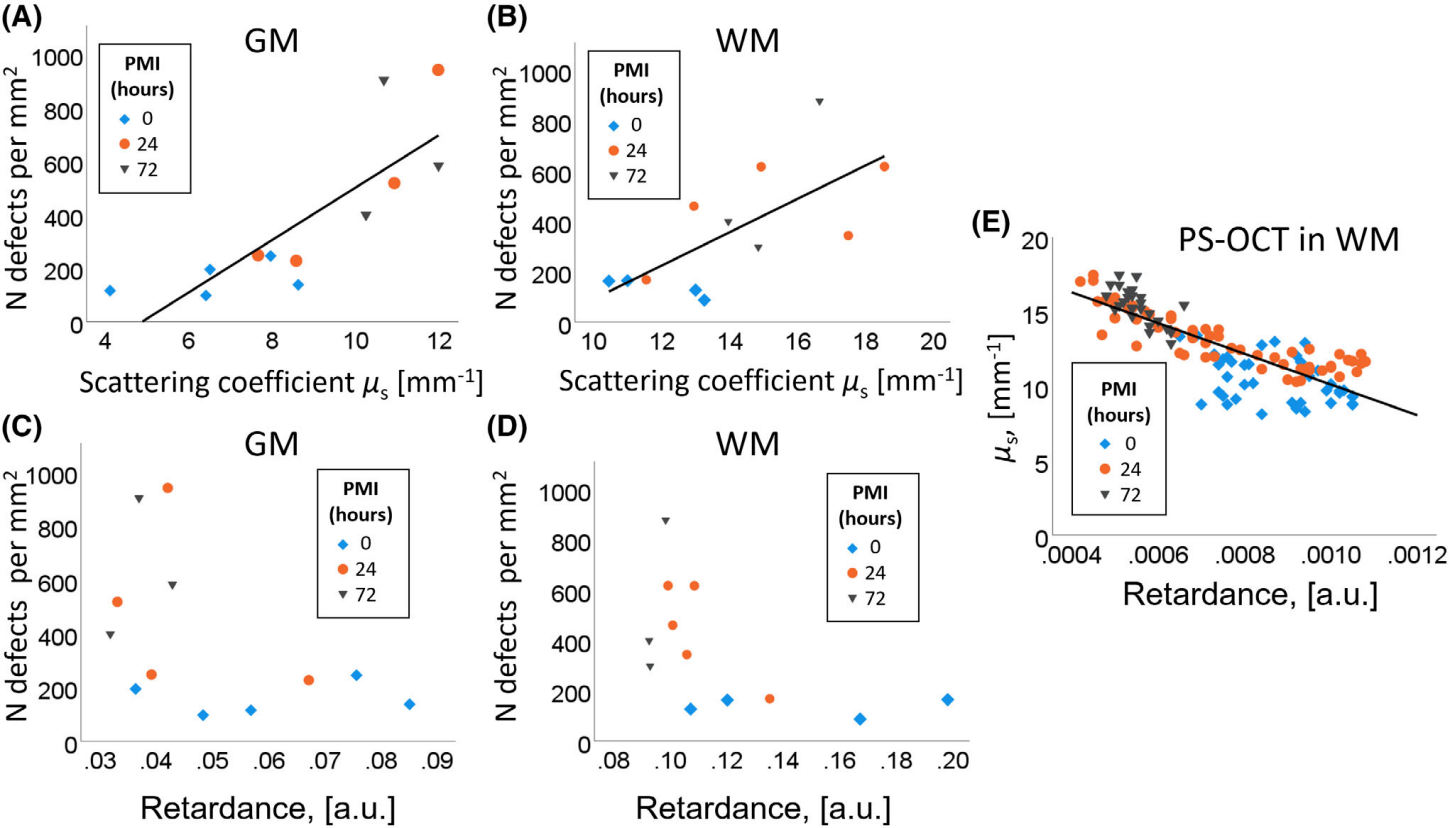
Correlations between optical properties and myelin defects. (A) Positive correlation between scattering coefficient and total defect counts in gray matter (*r* = 0.676, *p* = 0.016) and (B) white matter (*r* = 0.741, *p* = 0.006). (C) Negative correlation between quantitative birefringence microscopy relative retardance and defect counts in gray matter (*r* = −0.643, *p* = 0.024) and (D) white matter (*r* = −0.388, *p* = 0.212). (E) Negative correlation between polarization‐sensitive optical coherence tomography (PS‐OCT) relative retardance and scattering coefficient in white matter tissue (*r* = −0.558, *p* < 0.001). All correlations computed using Pearson method on residuals from linear mixed‐effects models controlling for postmortem interval (PMI) and animal ID. Data points colored by PMI show temporal progression of myelin defects.

## 4 DISCUSSION

This study provides the first quantitative characterization of PMI effects on myelin optical properties in primate brain tissue using polarization‐sensitive imaging techniques. Our results demonstrate that PMI produces progressive, quantifiable changes in myelin relative retardance and scattering properties detectable by PS‐OCT and qBRM. The systematic increases in scattering coefficients and decreases in relative retardance with longer PMI intervals establish these optical parameters as quantitative biomarkers of postmortem tissue degradation.

The observed changes align with our conceptual model of PMI‐induced myelin degradation (Figure 1). As myelin undergoes structural breakdown through lipid oxidation and protein denaturation processes [22, 23], the highly organized lamellar architecture becomes disrupted [24], creating numerous smaller particles that increase light scattering [25]. The strong positive correlations between scattering coefficient and defect density in both gray matter and white matter provide strong support for this biophysical interpretation, demonstrating that tissue‐level optical changes directly reflect the accumulation of microscopic structural disruptions.

The negative correlations between relative retardance and defect counts, together with the inverse relationship between scattering and relative retardance, support the premise that these optical parameters capture complementary aspects of myelin degradation. Scattering reflects particle‐level disruptions from fragmentation, while relative retardance reflects loss of organized structural anisotropy [26]. The strong correlation between scattering coefficient and relative retardance demonstrates that PMI‐induced myelin degradation simultaneously increases light scattering while reducing structural anisotropy, validating both optical parameters as complementary indicators of tissue integrity. This multi‐scale optical approach provides comprehensive characterization of PMI‐induced tissue changes.

The tissue‐specific distribution of defect types provides insight into regional vulnerabilities. The predominance of vesicles in gray matter suggests that myelin membrane structure in this region is particularly susceptible to vesiculation during postmortem degradation. In contrast, white matter showed a more balanced defect distribution, with a combination of swelling and delamination/blebbing defects comprising 36.7% alongside vesicles (35.1%). These regional differences likely reflect distinct myelin architecture and degradation pathways between gray and white matter, with gray matter's thinner, less densely packed myelin more prone to membrane fragmentation while white matter's thicker sheaths show greater structural defect diversity.

Axonal swelling is a recognized feature of postmortem tissue that can increase with both PMI and age. Because qBRM and PS‐OCT signals arise specifically from the birefringent myelin sheath rather than the axoplasm, our primary techniques cannot directly assess axonal status. In a limited qualitative assessment using dMBP/SMI312 immunolabeling (Supporting Information S3: Additional File 3), we did not observe axonal swelling co‐localizing with regions of myelin swelling; however, this observation was qualitative and limited in scope, and we cannot exclude a subtle indirect contribution of axonal changes—for instance, through stretching or displacement of the myelin sheath—to the optical signal changes observed here. Quantitative, co‐registered immunolabeling of axons and myelin across PMI timepoints represents an important direction for future work to more definitively address this question.

These physical insights complement existing literature on PMI effects across multiple scales. Ferrer et al. [7] demonstrated ultrastructural myelin changes with increasing PMI using electron microscopy, while D'Arceuil and de Crespigny [8] showed PMI‐dependent alterations in diffusion tensor imaging parameters, specifically decreased FA and increased mean diffusivity (MD) [27]—changes consistent with myelin degradation and loss of white matter integrity. Our findings position polarization‐based optical imaging as a mesoscopic bridge between ultrastructural and bulk neuroimaging measures, providing tissue analysis that directly relates to both microscopic changes and macroscopic properties. The temperature‐dependent degradation patterns reported in previous studies [24, 28] align with our controlled 4°C storage conditions, which likely slowed degradation compared to room temperature storage, suggesting that PMI effects under less controlled conditions could be more pronounced than observed here.

The convergence of findings across imaging modalities validates our optical approach while revealing important technical considerations. The divergent behavior of PS‐OCT relative retardance measurements in gray matter, where values increased rather than decreased with PMI, is consistent with previous reports demonstrating that in highly scattering media with dense cellular structures, increased scattering can overwhelm the relative retardance signal [29, 30]. This effect is specific to depth‐resolved techniques like PS‐OCT and was not observed in qBRM measurements of the same tissue. These observations highlight the importance of matching imaging modality and sample preparation to tissue type. PS‐OCT relative retardance measurements are dominated by myelin birefringence that overwhelms signals from oligodendrocytes and other cells in white matter, while qBRM performance in gray matter benefits from appropriate mounting media that closely matches brain tissue refractive index [21]—and thinner sections that minimize scattering artifacts.

These findings have immediate practical implications for postmortem optical imaging studies. The progressive nature of PMI‐induced changes demonstrates that myelin degradation artifacts can accumulate within 24 h postmortem, a timeframe common in tissue preservation protocols [31, 32]. Our data provide quantitative baselines that can aid in distinguishing PMI‐related artifacts from disease‐associated myelin pathology. The detection of significant effects within 24 h, becoming pronounced by 72 h, emphasizes the need for rigorous PMI documentation in study design. The quantitative relationships between PMI and optical properties enable development of correction factors and quality‐control thresholds for postmortem tissue studies, similar to approaches implemented in molecular research [33].

Importantly, many of the optical and morphological features altered by PMI overlap with those observed in true myelin pathology, including increased scattering, reduced birefringence, vesiculation, and lamellar disruption reported in demyelinating disease, aging, and white matter injury [10, 34]. Without quantitative baselines for PMI effects, such changes may be erroneously attributed to disease processes. Our findings, therefore, do not challenge the validity of myelin pathology studies, but rather provide essential context for distinguishing postmortem artifacts from real pathological alterations.

We acknowledge that our controlled experimental design does not fully represent real‐world tissue preservation conditions. We used exsanguinated samples (via Krebs buffer perfusion) and consistent 4°C storage, whereas actual tissue banking involves retained blood, variable temperature control, and variable in situ periods before extraction. Blood retention may accelerate degradation through hemolysis and oxidative processes [35] not present in our perfused samples, while temperature fluctuations could produce more severe degradation than our constant refrigeration [24]. *These factors suggest our findings represent conservative estimates of PMI effects*. The lower inter‐rater reliability in white matter (ICC = 0.443) compared to gray matter (ICC = 0.756) likely reflects the greater challenges of manual annotation in densely myelinated regions, where higher defect density and overlapping structures complicate comprehensive counting. The comparatively high proportion of mixed‐type defects reflects the current three‐category classification scheme's limited ability to capture the full diversity of degradation morphologies observed here; future work incorporating a broader defect taxonomy and/or automated classification approaches would likely reduce this ambiguity.

While our sample size of five animals enabled detection of large PMI effects with good statistical power, future studies with larger cohorts could examine individual variability and age‐dependent responses. Additionally, our focus on occipital lobe tissue limits generalizability to other brain regions that may show different vulnerability patterns.

Expanding these investigations across different brain regions, age groups, PMI intervals, and species will enable development of comprehensive guidelines for postmortem optical imaging. The heterogeneous spatial distribution of degradation observed here suggests that multiple sampling regions should be analyzed to obtain representative measurements. These optical methods offer opportunities to optimize preservation protocols by quantitatively assessing fixation and storage condition effectiveness. These approaches could form the basis for standardized quality‐control pipelines in postmortem neuroscience, potentially developing rapid quality assessment tools for research institutions.

## 5 CONCLUSIONS

PMI produces measurable, progressive changes in myelin optical properties within 24 h postmortem, becoming pronounced by 72 h, with scattering coefficients increasing and relative retardance decreasing in both gray and white matter. These changes correlate directly with accumulating morphological defects—vesicles, swellings, and structural discontinuities—visible at single‐axon resolution. Critically, these PMI‐induced features overlap substantially with those seen in genuine myelin pathology, underscoring the risk of misattribution in postmortem neuropathological studies conducted without rigorous PMI documentation. PS‐OCT and qBRM provide complementary, quantitative tools for detecting and characterizing this degradation, and the quantitative relationships between PMI and optical properties demonstrated here may support future development of quality‐control frameworks for postmortem optical imaging studies. Extending this framework to additional brain regions, species, and storage conditions will be essential for developing standardized guidelines applicable to human brain banking and archival tissue research in aging and neurological disease.

## AUTHOR CONTRIBUTIONS

**AN** and **RR** contributed equally to this work and share first authorship. **AN** performed polarization‐sensitive optical coherence tomography (PS‐OCT) and quantitative birefringence microscopy (qBRM) imaging, conducted statistical analysis, and was the primary author of the manuscript. **RR** prepared brain tissue for fixation and performed immunohistochemical staining. **PN** performed white matter qBRM imaging and myelin defect annotation. **AJ** conducted the preliminary study establishing the imaging pipeline. **TX**, **AT**, and **PN** performed myelin defect annotation. **AA** maintained and supported the PS‐OCT imaging system. **RN** and **YL** performed brain tissue preparation. **QY** advised on statistical methodology and mixed‐effects modeling approach. **FM** supervised immunohistochemical staining and contributed to manuscript preparation. **DB** supervised PS‐OCT physics and scattering coefficient (μ_s_) fitting methodology. **HW** supervised PS‐OCT imaging and contributed to backscattering (μ_b_) and scattering coefficient (μ_s_) fitting. **TLM** provided advisory oversight and performed surgical procedures on animal subjects. **DLR** supervised immunohistochemical staining and nonhuman primate work. **IJB** supervised qBRM methodology and optical physics. All authors read and approved the final manuscript.

## FUNDING INFORMATION

Research reported in this publication was supported by the National Institutes of Health, under Award Numbers R01 AG075727, R01 AG078460, and RF1 AG068168‐06. The content is solely the responsibility of the authors and does not necessarily represent the official views of the National Institutes of Health. The funders had no role in study design, data collection and analysis, decision to publish, or preparation of the manuscript.

## CONFLICT OF INTEREST STATEMENT

The authors declare that they have no competing interests.

## ETHICS STATEMENT

All animal procedures were approved by the Boston University Institutional Animal Care and Use Committee (IACUC protocol #00053) and conducted in accordance with the applicable laws and regulations governing the care and use of laboratory animals.

## Supporting information


**Data S1.** System validation.

**Data S2.** Cryosectioning protocol for white matter analysis.

**Data S3.** Immunohistochemical validation.

**Table S1.** Two‐way ANOVA: overall effect of PMI on defect counts.
**Table S2.** Post hoc pairwise comparisons between PMI timepoints.

## Data Availability

The datasets generated and/or analyzed during the current study are available from the corresponding author on reasonable request.
