# Supplementary material for: Effects of postmortem interval on myelin integrity in rhesus macaque brain tissue
Source: Brain Pathol. 2026 Oct 16:e70158. Online ahead of print. doi: 10.1111/bpa.70158 (PMC13675610; doi:10.1111/bpa.70158)
Supplement: Supplementary file 1 [file BPA-9999-e70158-s001.docx]

**Additional File 3**

**Immunohistochemical Validation**

**Figure 1**. Immunohistochemical labeling for axonal neurofilaments (SMI312, green) and degraded myelin basic protein (dMBP, red) at 0h (left), 24h (center), and 72h (right) PMI, imaged using a Leica TCS SP8 confocal microscope (40×); image stacks processed and rendered in Leica LAS X software. Tissue shows progressive structural disruption across the PMI, with marked disorganization between 0h and 24h, and further degradation by 72h. Staining becomes increasingly nonspecific with PMI duration, with reduced uptake into discrete structures and larger diffuse aggregates of label at later timepoints. Images shown as merged composites; green: SMI312, red: dMBP. Scale bar: 50 μm.
