# Supplementary material for: Effects of postmortem interval on myelin integrity in rhesus macaque brain tissue
Source: Brain Pathol. 2026 Oct 16:e70158. Online ahead of print. doi: 10.1111/bpa.70158 (PMC13675610; doi:10.1111/bpa.70158)
Supplement: Supplementary file 2 [file BPA-9999-e70158-s002.docx]

**Additional File 4**

**Sensitivity Analysis: Two-Way ANOVA with Tukey’s HSD and Bonferroni Post-Hoc Comparisons**

Defect counts shown in Fig. 6d,e (gray matter) and Fig. 7d,e (white matter) were re-analyzed using a two-way ANOVA (animal ID and PMI as fixed factors) with Tukey’s HSD and Bonferroni-corrected post-hoc pairwise comparisons, as an alternative to the primary linear mixed-effects model (LMM) reported in the main text. This analysis was performed using IBM SPSS Statistics.

**Table S1. Two-way ANOVA: overall effect of PMI on defect counts**

| **Region** | **Defect type** | **Levene’s test (variance homogeneity)** | **PMI effect: F (df1, df2)** | **PMI effect: p** |
| --- | --- | --- | --- | --- |
| Gray matter | Vesicle | F=4.85, p<.001* | F(2,93)=9.12 | <.001 |
| Gray matter | Swelling/delamination | F=1.72, p=.088 | F(2,93)=5.78 | .004 |
| White matter | Vesicle | F=1.64, p=.102 | F(2,93)=10.04 | <.001 |
| White matter | Swelling/delamination | F=3.11, p=.001* | F(2,93)=18.73 | <.001 |

**Levene’s test indicates unequal variances across groups for gray matter vesicles and white matter swelling/delamination defects; ANOVA results for these two measures should be interpreted with this in mind.*

**Table S2. Post-hoc pairwise comparisons between PMI timepoints**

| **Region** | **Defect type** | **Comparison** | **Tukey’s HSD p** | **Bonferroni p** |
| --- | --- | --- | --- | --- |
| Gray matter | Vesicle | 0h vs 24h | <.001 | <.001 |
|  |  | 0h vs 72h | <.001 | <.001 |
|  |  | 24h vs 72h | .050 | .058 |
| Gray matter | Swelling/delamination | 0h vs 24h | .041 | .047 |
|  |  | 0h vs 72h | <.001 | <.001 |
|  |  | 24h vs 72h | .002 | .002 |
| White matter | Vesicle | 0h vs 24h | <.001 | <.001 |
|  |  | 0h vs 72h | <.001 | <.001 |
|  |  | 24h vs 72h | .988 | 1.000 |
| White matter | Swelling/delamination | 0h vs 24h | <.001 | <.001 |
|  |  | 0h vs 72h | <.001 | <.001 |
|  |  | 24h vs 72h | .832 | 1.000 |

Every pairwise comparison between 0 h and a later PMI timepoint (0 h vs. 24 h; 0 h vs. 72 h) — the comparisons underlying the findings reported in the main text — remained statistically significant under both Tukey’s HSD and Bonferroni correction, in every region and defect type.
