# Supplementary material for: Effects of postmortem interval on myelin integrity in rhesus macaque brain tissue
Source: Brain Pathol. 2026 Oct 16:e70158. Online ahead of print. doi: 10.1111/bpa.70158 (PMC13675610; doi:10.1111/bpa.70158)
Supplement: Supplementary file 3 [file BPA-9999-e70158-s003.docx]

**Additional File 1**

**System Validation**

Quantitative assessment of PMI effects on myelin integrity requires precise optical measurements capable of detecting subtle biological changes above measurement noise. Before applying PS-OCT to tissue analysis, we established measurement reliability using controlled phantoms to answer three critical questions: (1) What is the inherent precision of our optical measurements? (2) Are we sensitive enough to detect PMI-induced changes? (3) Does our complete sample preparation workflow introduce systematic errors?

To address these questions systematically, we employed both computational modeling and experimental validation. Computational error analysis was first conducted to identify the primary sources of measurement uncertainty and optimize acquisition parameters, followed by phantom validation experiments to confirm theoretical predictions under controlled conditions.

**1 Computational Error Analysis**

**1.1 Model Parameters**

Prior to experimental validation, we conducted comprehensive computational error analysis (N = 1000 iterations) to characterize measurement uncertainty and identify optimal acquisition parameters. The simulations modeled the PS-OCT signal equation[1]:

$$R\left( z \right)=I_{0}+\sqrt{\mu_{b}\cdot e^{-2\mu_{s}z}\cdot\frac{1}{1+\left( \frac{z-z_{f}}{z_{r}} \right)^{2}}}, (S1)$$

where R is the reflectance signal, z is depth, μₛ is the scattering coefficient, μᵦ is the backscattering coefficient, z_f_ is the focus position, zᵣ is the Rayleigh range, and I₀ is the intensity offset.

Four primary sources of measurement uncertainty were systematically evaluated:

1. **Gamma speckle noise effects**: Gamma-distributed noise was modeled to represent the well-established multiplicative speckle noise characteristic of OCT systems, as demonstrated by Varadarajan et al. (2022) in their comprehensive analysis of ex vivo human brain OCT images[2]. The speckle noise follows a gamma distribution and significantly degrades the contrast-to-noise ratio between tissue structures, making accurate quantitative analysis challenging.
2. **Focus position (z_f_) estimation errors**: Random noise (0 to 50 μm) was added to the true focus position to simulate focus positioning uncertainty during calibration.
3. **Rayleigh range (zᵣ) estimation errors**: Random noise (0 to 10 μm) was introduced to the confocal parameter to assess sensitivity to beam waist variations estimation.
4. **Fitting depth optimization**: Fit depths ranging from 50-180 pixels were tested to determine the optimal signal analysis window.

For each scenario, percentage errors were calculated as 100 × (measured value – actual value)/actual value, and statistical measures including standard deviation and median were computed to characterize measurement uncertainty.

**1.2 Simulation Results**

**Table 1. Percentage Error Results from Computational Error Analysis**

| **Parameter** | **Mean±SD, %** | **Min to Max, %** | **Median, %** |
| --- | --- | --- | --- |
| Gamma noise | 6.8 ± 7.8 | -19 to 52 | 5.3 |
| z_f_ noise | 32 ± 88 | -6 to 1001 | 5.5 |
| zᵣ noise | -2.2 ± 2.6 | -8.7 to 26.8 | -2 |

**Key simulation findings:**

- Gamma speckle noise contributes ±7% error across all μₛ ranges tested, consistent with the multiplicative speckle characteristics documented by Varadarajan et al. (2022) [2]
- Gamma-distributed speckle noise tends to systematically overestimate μₛ values
- Focus position (z_f_) estimation error was identified as the largest contributor to measurement uncertainty
- Optimal fit depth was determined to be above 100 pixels

**Figure 1.** Computational error analysis results (N=1000). (a) Percentage error across different scattering coefficients from gamma noise influence; (b) influence of incorrect determination of zᵣ value; (c) influence of incorrect z_f_ value determination; (d) fitting depth optimization, showing error rate minimization after 100 pixel fitting depth.

These simulation results directly informed the experimental design, particularly the emphasis on precise focus positioning and standardized fitting protocols.

**2. Phantom Preparation and Validation Protocols**

**2.1 Calibration TiO₂ Agar Preparation**

Based on simulation requirements for a stable calibration reference, we developed a standardized protocol for preparing titanium dioxide (TiO₂) agar phantoms. The TiO₂ agar serves as a scattering reference with known optical properties (μₛ = 6.0 mm⁻¹), enabling separation of instrument-dependent parameters from tissue-specific optical properties during the fitting procedure.

**Materials (per 50ml):**

- 50ml 1× PBS buffer (pH 7.4)
- 0.25g titanium dioxide powder (TiO₂)
- 2.25g agarose powder (low melting point)
- 50μl Tween-20 surfactant

**Preparation Protocol:**

1. Mix 1ml PBS with 0.25g TiO₂ using spatula to form homogeneous paste
2. Slowly add remaining 49ml PBS with vigorous mixing to prevent aggregation
3. Add 50μl Tween-20 and magnetic stir for 1 minute
4. Sonicate mixture for 5 minutes to eliminate particle clusters
5. Add 2.25g agarose powder and magnetic stir for 3 minutes
6. Sonicate again for 5 minutes to ensure complete homogenization
7. Microwave at 700W for 1 minute until agarose completely dissolves
8. Allow slight cooling (to ~60°C) before pouring to prevent thermal damage
9. Pour into molds and refrigerate at 4°C for solidification

This protocol consistently produces agar with μ_s_ = 6.0 ± 0.3 mm⁻¹, providing the calibration matrix used throughout all measurements.

**Figure 2. Phantom validation and measurement repeatability.** (a) Validation phantom containing polystyrene bead regions with controlled scattering coefficients (0-14 mm⁻¹) embedded within TiO₂ agar matrix (6 mm⁻¹). Yellow boxes indicate measurement regions for different measurement trials. Blue and white boxes show measurement regions to evaluate phantom homogeneity. This design replicates tissue imaging workflow where brain samples are embedded in calibration agar (b). (c) A-line profiles across different focus depths (z_f_) in highly scattering media (μ_s_ = 16 mm⁻¹), showing maximum signal when z_f_ = 0 at tissue surface. (d) A-line profiles for embedding agar (μ_s_ = 6 mm⁻¹), demonstrating how fitting profile shape changes with focusing depth, enabling accurate z_f_ calibration during Step 1 parameter extraction.

**2.2 Validation Phantom Design**

To replicate the tissue imaging workflow, validation phantoms were prepared by embedding blocks with various scattering coefficients (0-14 mm⁻¹) within the TiO₂ agar matrix (6 mm⁻¹ scattering) (see **Fig. S2a**). This design mimics the actual tissue imaging protocol where multiple brain samples with different PMI intervals are embedded within the TiO₂ agar block (**Fig. S2b**).

Different scattering coefficients in phantom regions were achieved by varying polystyrene microsphere concentrations (1 μm diameter) in agarose. Target regions were created with μ_s_ values from 0 to 14 mm⁻¹ by adjusting polystyrene beads density. Each region was formed in separate molds, then carefully embedded within the 6 mm⁻¹ TiO₂ agar to create distinct measurement zones.

**3. Optical Property Extraction Methodology**

**3.1 Two-Step Fitting Approach**

Scattering coefficient extraction follows a two-step fitting approach that ensures accurate quantification across different tissue types and experimental conditions by separating instrument-dependent parameters from tissue-specific optical properties. This approach addresses the fundamental challenge that the OCT signal equation (S1) contains both unknown tissue properties and unknown system parameters that cannot be reliably determined simultaneously from tissue measurements alone.

**Step 1: System parameter calibration using agar phantom.** Optical system parameters were extracted from the known 6 mm⁻¹ TiO₂ agar to establish baseline measurements for subsequent tissue analysis. We manually selected tiles containing only the TiO₂ agar phantom while avoiding polystyrene bead regions or tissue samples (**Fig. S2b**), then averaged across multiple TiO₂ agar measurements to reduce noise and improve parameter extraction reliability.

The combined A-line signals from the 3D agar volume were fitted simultaneously to the OCT model by equation (S1), where μ_s_ = 6.0 mm⁻¹ was fixed as the known scattering coefficient of the calibration agar, while μ_b_ (backscattering coefficient), z_r_ (Rayleigh range/confocal parameter), and I₀ (intensity offset) were fitted as single values across the entire field of view. Critically, z_f_(x,y) representing focal depth variations across the imaging field was fitted as a spatially-varying parameter and subsequently mapped using a 2D polynomial: z_f_(x,y) = Σᵢⱼ aᵢⱼ × xⁱ × yʲ. Parameter extraction quality was validated by requiring R² > 0.98 for acceptable calibration.

**Step 2: Tissue/sample property extraction.** Calibrated system parameters were applied to extract unknown optical properties from tissue or validation phantom regions by fixing the instrument-dependent parameters and solving for tissue-specific properties. We loaded the calibration parameters z_r_, z_f_(x,y), and I₀ determined in Step 1, then fitted each individual A-line in tissue or polystyrene phantom regions to the same OCT model equation (S1).

**3.2 Surface Detection and Focus Positioning Protocol**

Highly scattering agar regions (μ_s_ = 16 mm⁻¹) were used to locate tissue surface, where A-line signal drops as focus position shifts from the surface (**Fig. S2c**). Focus was positioned 120 μm below detected surface for optimal fitting conditions. The 6 mm⁻¹ scattering agar provides optimal signal shape for parameter fitting while allowing clear differentiation of focal depth variations (z_f_) during calibration (**Fig. S2d**). Higher scattering regions (16 mm⁻¹) enable precise surface detection but would saturate the fitting algorithm if used for calibration.

**4. Measurement Repeatability and Validation Results**

**4.1 Repeatability Assessment Protocol**

Three independent measurements were performed across six regions over 24-hour intervals under the same environmental conditions, with complete system shutdown and restart between each measurement session. This protocol required full recalibration including focus positioning and imaging parameter setup for each trial, ensuring assessment of complete system reproducibility rather than within-session repeatability alone.

Measurement repeatability was quantified using intraclass correlation coefficient (ICC) analysis with a two-way random effects model. Effect sizes were quantified using Cohen's d, calculated from pooled standard deviations within each tissue type. Statistical power analysis employed standardized effect sizes with n=5 animals per group.

**4.2 Validation Outcomes**

Phantom validation demonstrated excellent measurement repeatability across six controlled scattering regions over three independent trials. Intraclass correlation coefficient analysis using a two-way mixed effects model yielded ICC = 0.951 (95% CI: 0.569-0.993), indicating that 95.1% of measured variance reflected true regional differences rather than measurement error. Coefficients of variation ranged from 9.2-17.0% across regions with adequate signal (average CV = 12.5%).

Within-subject standard deviation of 1.12 mm⁻¹ represents the typical measurement uncertainty for repeated measurements on the same sample, confirming adequate measurement precision for detecting PMI-induced changes**.**

**4.3 Spatial Uniformity Assessment**

Spatial uniformity within phantom regions was assessed by performing multiple measurements (n=12-14) at different locations within selected regions to distinguish measurement variability from material inhomogeneity. Spatial coefficients of variation (4.5-12.0%) were comparable to or lower than temporal repeatability measures, validating the use of phantom regions as controlled standards for system calibration.

**4.4 Implementation Guidelines**

The protocols validated here enable implementation across different PS-OCT systems and research settings. Key requirements for reproducibility include: (1) standardized tissue embedding procedures maintaining calibration agar reference in all measurements, (2) implementation of the two-step fitting approach with proper calibration requiring R² > 0.98 for system parameter extraction, and (3) consistent focus positioning protocols using high-scattering reference regions for surface detection.

**5 Conclusion**

The demonstrated measurement precision (ICC = 0.951, CV = 12.5%) establishes optical imaging as a reliable tool for quantifying PMI effects in postmortem studies. Computational error analysis identified focus positioning accuracy as the critical factor determining measurement precision, emphasizing the importance of standardized surface detection protocols.

**Supplementary References**

[1]	J. Yang *et al.*, “Improving the characterization of ex vivo human brain optical properties using high numerical aperture optical coherence tomography by spatially constraining the confocal parameters,” *Neurophotonics*, vol. 7, no. 4, p. 045005, Oct. 2020, doi: 10.1117/1.NPH.7.4.045005.

[2]	D. Varadarajan, C. Magnain, M. Fogarty, D. A. Boas, B. Fischl, and H. Wang, “A novel algorithm for multiplicative speckle noise reduction in ex vivo human brain OCT images,” *Neuroimage*, vol. 257, p. 119304, Aug. 2022, doi: 10.1016/J.NEUROIMAGE.2022.119304.
