# Supplementary material for: Effects of postmortem interval on myelin integrity in rhesus macaque brain tissue
Source: Brain Pathol. 2026 Oct 16:e70158. Online ahead of print. doi: 10.1111/bpa.70158 (PMC13675610; doi:10.1111/bpa.70158)
Supplement: Supplementary file 4 [file BPA-9999-e70158-s004.docx]

**Additional File 2**

**Cryosectioning Protocol for White Matter Analysis**

**Figure 1.** Step-by-step illustration of the cryosectioning workflow used to obtain 7 μm brain sections following PS-OCT imaging. **(1)** Preparation of OCT compound base in NinjaFlex mold at -20°C. **(2)** Trimming of OCT surface to ensure parallel alignment with cutting plane. **(3)** Placement of 150 μm brain slice on polyethylene film with excess sucrose removal. **(4)** Transfer of tissue to prepared OCT chuck using gentle pressure to ensure adhesion. **(5)** Complete embedding in OCT compound and final sectioning at desired thickness. This protocol ensures optimal tissue preservation and section quality for subsequent BRM imaging while minimizing artifacts from processing.

**Detailed Protocol:**

1. **Prepare OCT base:** Pour OCT compound into a 30 mm NinjaFlex mold on a cryostat chuck and freeze at approximately -20°C inside the cryostat
2. **Surface preparation:** Remove mold, place chuck into holder with proper notch alignment, and trim OCT base surface flat using cryostat blade to ensure parallel positioning to cutting plane
3. **Tissue preparation:** Place 150 μm brain slice on polyethylene (PE) film tissue-side up, and gently remove excess sucrose solution using lens cleaning tissue
4. **Sample transfer:** Press prepared chuck onto tissue while minimizing time outside cryostat, ensuring even contact without sample damage. Gently tap base to promote tissue adhesion
5. **Final embedding:** Reapply NinjaFlex mold, add additional OCT compound to fully embed sample, and freeze newly added OCT in cryostat chamber
6. **Sectioning:** Place chuck in holder with same alignment as preparation, trim OCT until tissue surface is reached, section at desired thickness, and mount on microscope slides.
